# Spotlight on clinical strategies of Chronic Internal Carotid Artery Occlusion: Endovascular interventions and external-intracarotid bypasses compared to conservative treatment

**DOI:** 10.3389/fsurg.2022.971066

**Published:** 2022-11-08

**Authors:** Junnan Wu, Chaoyou Fang, Lingying Wei, Yibo Liu, Houshi Xu, Xiaoyu Wang, Ling Yuan, Xiaoya Wu, Yuanzhi Xu, Anke Zhang

**Affiliations:** ^1^ Department of Emergency, Dongyang Hospital Affiliated to Wenzhou Medical University, Jinhua, China; ^2^ Department of Neurosurgery, Shanghai General Hospital, School of Medicine, Shanghai Jiao Tong University, Shanghai, China; ^3^ Department of Neurosurgery, The Second Affiliated Hospital, School of Medicine, Zhejiang University, Shanghai, China; ^4^ Clinical Research Center for Neurological Diseases of Zhejiang Province, Hangzhou, China; ^5^ Department of Neurosurgery, Huashan Hospital, School of Medicine, Fudan University, Shanghai, China

**Keywords:** chronic internal carotid artery occlusion, endovascular interventional therapy, external carotid-internal carotid bypass, review, comparison

## Abstract

Chronic internal carotid artery occlusion (CICAO) has high prevalence and incidence rates, and patients with CICAO can be completely asymptomatic, experience a devastating stroke or die. It is important to note that CICAO causes cerebrovascular accidents. Currently, the external carotid-internal carotid (EC-IC) bypass technique is used to treat CICAO. However, many clinical studies showed that EC-IC bypass was not beneficial for many patients with CICAO. Meanwhile, endovascular intervention treatment options for CICAO are evolving, and an increasing number of patients are undergoing endovascular intervention therapy. Accordingly, a review comparing both techniques is warranted. For this review, we searched PubMed and collected relevant case study reports comparing endovascular interventional therapy and internal and external cervical bypass surgeries to provide strategies for clinical treatment.

## Introduction

Internal carotid artery occlusion (ICAO) can be classified as acute internal carotid artery occlusion (AICAO) and CICAO (1–4). Normally, complete internal carotid occlusion that lasts more than 4 weeks is considered CICAO (5). The main causes of CICAO include carotid atherosclerotic disease, heart-derived thrombosis, and carotid artery dissection (6, 7). Previous studies revealed that CICAO is usually a result of complex hemodynamics, such as low shear stress, blood flow stasis, and blood flow separation (8, 9). In addition, radiation therapy may cause chronic occlusion of the carotid artery (10). Of note, occlusion caused by carotid dissection mainly occurs in young individuals, and the pathogenic site is often at the entrance of hard vessels (11). In elderly individuals, occlusion is usually caused by atherosclerosis, which generally occurs proximal to the internal carotid artery. Additionally, CICAO caused by atherosclerotic plaque of the stenotic carotid artery is the underlying cause of major ischemic strokes (12).

Antiplatelet aggregation therapy is currently the basic treatment for secondary prevention of ischemic cerebrovascular disease. Low-dose aspirin (75–150 mg) is effective for secondary prevention of minor stroke or TIA episodes and can significantly reduce the risk of vascular events (13). However, healthy lifestyle changes, including healthy diet and appropriate exercising can reduce the risk of stroke with no need for additional medical intervention in partial patients with CICAO. Most symptomatic patients require an urgent visit to the hospital for treatment (14). In terms of drug treatment, if there are no contraindications, it is recommended to take double antibody within 3 months after the onset, and then long-term monoclonal antibody platelet aggregation treatment (13). A previous study showed that patients receiving conservative treatment for CICAO-induced stroke had poor outcomes (15). In addition, Robert et al. also mentioned that with medical treatment alone, the risk of ipsilateral ischemic stroke 2 years after carotid occlusion is still 5%–8% per year (16). Therefore, while medication can reduce the risk of stroke, it does not eliminate the risk entirely (17). The combination of conservative treatment and surgical intervention may be an effective approach for patients with CICAO. With the development of clinical management, there are various approaches to treat symptomatic patients with CICAO, such as conservative treatment, surgical EC-IC bypass, endovascular interventional therapy, carotid endarterectomy, and hybrid surgery (14, 18).

Currently, surgical revascularization (EC-IC bypass) improves cerebral hemodynamics and oxygen extraction fraction and is used as a primary treatment option for patients with CICAO (19, 20). However, a large-scale cohort study showed that most patients with CICAO are not suitable for EC-IC bypass (21, 22). In addition, several studies of patients treated with endovascular or hybrid therapy have shown positive outcomes, which reflects the clinical value of either treatment. In this review, we summarize these treatment modalities based on the current data and evidence, compare the treatment effects of EC-IC bypass and endovascular treatment, and provide a reference for the choice of clinical treatment modalities.

## Epidemiology of CICAO

The clinical manifestations of CICAO vary widely; patients may be completely asymptomatic, experience a devastating stroke or die (23). An epidemiological study based on Americans showed that the incidence of ICAO is approximately 6 per 100,000, and approximately 60,000 cases of first stroke are diagnosed as ICAO (24, 25). The severity of the disease depends on the collateral circulation status, and well-compensated patients may be asymptomatic. Nevertheless, approximately 2%–8% of patients progress from asymptomatic occlusion to symptomatic occlusion every year, with an annual stroke rate of 4.4% and an annual transient ischemic attack (TIA) rate of 3.2% (26, 27). In addition, the annual stroke rate of symptomatic carotid artery obstruction is 6%–20% (28, 29), and of course, spontaneous re-channeling of the blood vessels may occur in some patients, accounting for approximately 2.3%–10.3% of patients (26).

## Underlying pathophysiological mechanisms

Intravascular hemodynamics depend largely on the anatomy of the vessel wall. The involvement of vessel wall mechanics and hemodynamics in atherosclerosis has been amply demonstrated (30). The vascular intimal layer, which is composed of endothelial cells, an internal elastic layer and fibrous collagen tissue, plays a key role in preventing platelet aggregation and antithrombosis (31, 32). Disruption of the endothelium is a key factor in the initial development of atherosclerotic disease within the vessel wall (33). After the endothelial layer is damaged, endothelial function is damaged, which promotes platelet adhesion and endothelial cell proliferation by releasing cytokines, which leads to hardened intima in the vascular lumen and causes the occurrence of atherosclerotic disease (34, 35). In addition, damage to the endothelium also causes structural changes to the media layer. Structural changes to the media layer occur through inflammatory stress and smooth muscle hyperplasia, thus causing fat stripes to form and ultimately leading to atherosclerosis (36–38). Over time, these changes and developments will gradually cause vascular lumen stenosis or even occlusion.

Different blood vessel diameters, endothelial thicknesses and blood vessel bifurcations easily cause blood turbulence. The uneven distribution of blood flow can easily cause local thrombosis, which is another cause of vascular occlusion (39). Therefore, additional carotid artery lesions are better developed at the carotid artery bifurcation (40). In summary, the pathogenesis of CICAO can be summarized in two aspects: the destruction of vascular endothelial cells causes local or other sources of thrombosis caused by atherosclerosis and blood turbulence.

## Clinical manifestations

The clinical symptoms of CICAO vary widely (41–44). If the collateral circulation is good, then the patient may have no clinical symptoms after ICAO (45). However, CICAO can also present as a transient ischemic attack, a focal stroke, or a massive cerebral infarction that results in patient death. Most patients with CICAO do not have any clinical symptoms (27). When intracranial hemodynamic changes induced by events, including postural transient ischemic attack, postprandial hypotension, heart failure, allergy, blood loss, dehydration, exercise and other factors, that lead to a decrease in cerebral perfusion pressure, then transient neurological deficiency symptoms will appear, and furthermore, a decrease in cerebral perfusion pressure may promote the occurrence of ischemic stroke symptoms. Very few patients with CICAO exhibit episodic involuntary movements of the ipsilateral limb and are often misdiagnosed as partial epilepsy. An electroencephalogram (EEG) is usually normal in such patients and a thermal conductivity detector (TCD) shows decreased cerebral blood flow. Patients with extremely rare CICAO present with decreased monocular vision under light variations ranging from dark to high light, possibly with increased retinal metabolic activity under light variations ranging from dark to high light, and no corresponding increase in cerebral blood flow, thus resulting in retinal ischemia. Paroxysmal involuntary movement of the ipsilateral limb or transient decline in monocular vision was highly suggestive of CICAO (23).

Patients with partial chronic occlusion of the internal carotid artery present with an unusual headache, due to compensation from the external carotid artery collateral circulation of the mandibular angle, eyebrow arch and buccal artery stroke, which is called ABC beating (41). With compensation from Willis' circle, no ABC pulsation was observed. The most common symptom of acute retinal ischemia is amaurosis fugax, and the most common cause of acute retinal ischemia is the loss of the embolus in the internal carotid artery. Chronic ocular ischemia syndrome with the clinical manifestations of a progressive decline in visual acuity is likely to develop in 4%–18% of patients with severe stenosis or occlusion of the internal carotid artery (42). Fundoscopy showed patchy retinal hemorrhage, arterial stenosis, and venous dilatation, and the late manifestations were flocculated exudation and angiogenesis. Some patients with CICAO present with transient syncope (43). Hypoperfusion associated with CICAO can even lead to vascular dementia (44). Some patients with chronic internal carotid artery occlusion (ICAO) develop neurological deficits in the future (46).

## Surgical therapeutic strategy

With the development of clinical management, there are various surgical approaches to treat symptomatic patients with CICAO, such as EC-IC bypass, endovascular interventional therapy, carotid endarterectomy, and hybrid surgery (47, 48).

### EC-IC bypass surgery

In the 1960s, the benefits of EC-IC bypass began to be recognized (49). At the same time, it is widely believed that EC-IC bypass can successfully treat ICAO to prevent cerebrovascular ischemia. Therefore, efforts to develop and improve some bypass techniques can lead to a sharp increase in the number of surgeries performed using the new and improved techniques. However, several research studies published in 1985 showed that there was no significant difference between EC-IC bypass surgery combined with drug therapy and optimal drug therapy alone in terms of reducing the risk of long-term stroke (21). However, this procedure continues to be reported and is beneficial in patients with a specific carotid or middle cerebral artery occlusion (50).

At present, the surgical methods are divided into high flow and low flow, and the main difference lies in the choice of the blood vessels that will receive surgical intervention (51). Low-flow surgery was performed on the superficial temporal artery-middle cerebral artery (STA-MCA), superficial temporal artery-superior cerebellar artery (STA-SCA), and ophthalmic artery-posterior inferior cerebellar artery (OA-PICA). However, STA-MCA, as the basic bypass for low-flow revascularization in neurosurgery, can only provide up to 1/4—1/3 of the normal MCA blood flow (52). STA-MCA provides an average blood flow of 30 ml/min shortly after the start of surgery, which can then gradually increase but can rarely reach the blood supply of the MCA (53, 54). Sometimes, to increase the blood supply, double bypass surgery is performed in the anterior temporal artery and the MCA (54). High-flow surgical methods include large saphenous vein transplantation and ulnar artery transplantation. Although high-flow surgical techniques can provide more blood flow, these methods are mostly used to treat a brain tumor or brain aneurysm, and few cases of carotid artery occlusion treated with these techniques have been reported.

EC-IC bypass is a new procedure to prevent and treat cerebral ischemia. At that time, blood vessels were reconstructed to treat cerebral ischemic diseases caused by vascular stenosis or occlusion. When the results of the EC-IC research group were published in 1985, the number of EC-IC bypass operations performed worldwide plummeted to only a few a year. However, some cerebrovascular surgeons have questioned the rationality, rigor, and statistical method of the study (55, 56) and have been using this surgery to prevent the occurrence of stroke. The controversies are as follows: (1) The inclusion criteria for the patients are controversial. The selected patients did not strictly regulate the compensation of the circulation after the evaluation of vertebral artery angiography, the intracranial collateral circulation before the selection, the cerebrovascular reserve capacity (CVR), or the decline in cerebral blood flow and metabolic disorders in the corresponding area. It is impossible to distinguish whether it is caused by embolus distal blood vessel embolization or cerebral blood supply disorder caused by hemodynamic reasons. Embolization was painful for patients undergoing cooperative EC-IC bypass (55, 56). (2) The number of patients selected is also controversial. The research team includes professionals from more than 100 medical centers in North America, Europe and Asia Pacific, and patients are prospectively randomized to internal medicine (714 for aspirin) and surgical treatment (663). However, the survey found that a large number of qualified patients in the surgical group were not included, which is likely to affect the accuracy of the study (55, 56).

Studies on EC-IC bypass are still ongoing in the 21st century. In 2006, the Japanese EC-IC Trial (JET) Study Group designed a pilot trial to evaluate the role of EC-IC bypass in preventing ischemic stroke recurrence in patients with major cerebral artery occlusive disease and hemodynamic cerebral ischemia (57). The trial randomly evaluated 196 patients (98 receiving maximal drug treatment; 98 undergoing EC-IC bypass + maximal drug treatment) who were followed up for 2 years. Preliminary data showed that the incidence of stroke recurrence in the surgical treatment group was significantly lower than that in the medical group (5% vs. 14%, *p*-value = 0.046). In 2011, the Carotid Occlusion Surgery Study (COSS) conducted a prospective, randomized trial that divided patients into groups A and B. Group A underwent STA-MCA bypass surgery combined with drug therapy, and Group B received the best drug therapy. However, the trial was terminated early because previous data showed no significant difference in the recurrence rate between groups A and B (58). Later, the Cerebrovascular Section of the American Association of Neurological Surgeons (AANS) and the Congress of Neurological Surgeons (CNS) reported that the COSS was flawed. Thus, the findings of the COSS are also controversial. They believe that EC-IC bypass surgery is still feasible for specific patients in technologically advanced medical centers (59). A similar assessment was published by the European Association of Neurological Surgeons (60).

Fiedler et al. showed a significant improvement in some neurocognitive function in 30 patients at 12 months before and after EC-IC bypass surgery (61). With the results of the randomized evaluation of Carotid Occlusion and Neurocognition (RECON) trial published, the effect of recovery of cognitive function has again been controversial. They showed no difference in the 2-year follow-up of neurocognitive function between the 13 EC-IC bypass surgery groups and the 16 drug treatment groups (62). However, the small sample size of the trial limits the reliability of the conclusions. At present, EC-IC bypass surgery is still considered the first treatment option for CICAO (19). However, large-scale cohort studies revealed the limited benefit of the EC-IC bypass procedure for patients with CICAO (21, 22). Therefore, there is an urgent need to investigate novel therapeutics with better outcomes for CICAO.

### Endovascular intervention

Endovascular treatment is also considered to be effective for patients with CICAO. Endovascular treatment includes carotid stenting (CAS) alone or the combination of carotid endarterectomy (CEA) and CAS (63–66). According to previous studies, the assessment of revascularization is mainly done by imaging, for example, high-resolution magnetic resonance vessel wall imaging (MR-VWI) and hemodynamic evaluation (67). Occlusion with the residual lumen and shorter occlusion length on high-resolution MR-VWI were identified as predictors of technical success of endovascular recanalization for nonacute ICAO (68). In addition, the evaluation of cerebral hemodynamics following recanalization therapy can also help identify patients at high risk for complications associated with reperfusion. CAS refers to the use of a micro guide wire to place a stent at the stenosis or occlusion site, which then expands through the stent to support the vessel and recanalize the blood flow. In hybrid surgery, CEA is performed at the initial site of the ICA and then at the distal end of the ICA, which is where the micro guide wire and micro guide catheter are placed. Hybrid surgery is relatively safe because by cutting the blood vessel, the thrombus debris of the neck blood vessels can be effectively removed, thus helping to prevent distal embolization accidents. Through these techniques, revascularization can be achieved through various degrees of vascular remodeling. According to some previous literature reviews, successful recanalization was defined as final residual diameter stenosis ≤20%. And the rate of successful recanalization varied from 60% to 100% according to previous literature (69).

We can adopt different endovascular treatment modalities depending on the location of the occluded vessels (70). If the occluded vessel is located in the skull, then the blood flow can be restored with an endovascular stent, which is the goal of treatment. If the occluded vessel is located in the neck, then the intima can be removed to restore the blood flow. Of course, some patients have occlusion vessels with long lengths that involve the neck and intracranial areas, and the guidewire may not successfully cross the occlusion site. At this time, we can choose hybrid surgery to achieve the purpose of treatment. In addition, the disadvantage of endovascular intervention is that the thrombus may be shed during balloon dilation or stent release, going distal to the blood flow, thereby blocking the intracranial artery (71, 72). Thus, experienced clinicians will protect the common and external carotid arteries in some cases (73, 74). Some doctors currently use the Parodi embolic protection system (75–77), but this method still has limitations. For example, if the thrombus aggressively occupies the blocked vessels, then the proximal occlusion may not necessarily provide additional protection (78).

In 2005, Terada introduced a new treatment method for CICAO patients, which uses the vascular recanalization technique (73). As interventional materials and technology advance, it has become more feasible and safer to open the occluded internal carotid artery. Patients with cervical ICA stenosis who undergo carotid endarterectomy (CEA) are protected against ischemic stroke, but not those with occlusions. However, when a patient is at high surgical risk and does not want to undergo CEA, endovascular carotid artery stenting (CAS) can be an alternative (79). Piotr's study also proposed that endovascular treatment for CICAO is safe and feasible, with a technical success rate of 67% and a low incidence of early and late neurological complications (80, 81). In recent years, an increasing number of research findings have been reported regarding the endovascular treatment of CICAO. In 2014, Fan et al. (82) conducted a prospective controlled study in which 40 patients were divided into two groups: A and B. Group A will receive endovascular treatment, and Group B will receive conservative treatment. The results showed that 16 of the 18 patients in group A had successful flow recanalization and no cerebrovascular events. Improvement in cerebral perfusion was observed in 12 patients according to single-photon emission computed tomography scans. At the 1-month, 3-month, and 6-month follow-ups, the Montreal Cognitive Assessment in Group A significantly outperformed Group B. Moreover, Xia et al. (83) also designed a controlled study of endovascular treatment and optimal drug treatment in the two groups, indicating that endovascular surgery had a good outcome in terms of neurological function. However, after two years of follow-up, there was no significant difference in the incidence of cerebrovascular accidents or mortality between the two groups. In the study by Lin et al., cases of stroke recurrence and death persisted after endovascular therapy (84). Therefore, whether endovascular therapy can be used as an effective treatment method still needs to be further proved by comparison.

Additionally, hybrid surgery has been reported successively in recent years, thus improving the success rate of endovascular surgical treatment (18, 85, 86). Shih et al. reported in 2013 that the surgery was performed in three patients with a recurrent ischemic attack and the patients achieved complete recanalization of blood flow (87). Improved cerebral perfusion was shown on computed tomography angiography and perfusion imaging. After 6 months of follow-up, the ischemic symptoms did not recur. This is an earlier reported success, but with a small sample size. In 2018, Zhang et al. (88) divided 65 patients with long ICA occlusion into revascularization hybrid surgery (*n* = 30) and medication (*n* = 35) groups to analyze clinical and angiographic data. The vascular recanalization rate of the patients who underwent hybrid surgery was 100%, with 2 complications in the perioperative period. One patient had a recurrent laryngeal nerve injury, and the other patient had an intracranial hemorrhage, but none of the patients developed severe neurological deficits, therefore suggesting that hybrid surgery may be safe and effective in achieving revascularization of long segment occlusion of the internal carotid artery to prevent further ischemic events.

## Comparison

Patients with transient ischemic attacks (TIA) and ischemic strokes are at high risk for stroke recurrence, especially companied with symptomatic internal carotid artery stenosis (89). The latest data using modern DAPT poses an important baseline by which to compare results with both endovascular and surgical strategies. It has been illustrated that timely initiation of effective antithrombotic therapy and revascularization interventions are urgently required to prevent the recurrent events (90). Perioperative aspirin presents a better outcomes in patients with ICA occlusion. Additionally, application of single antiplatelet therapy (SAPT) in patients with symptomatic carotid stenosis (Class I, Level A) was recommend by the American Heart Association/American Stroke Association (AHA/ASA) guidelines (91). Although the dual antiplatelet therapy (DAPT) with aspirin and clopidogrel is a more effective strategy than monotherapy to alleviate emboli and neurological events, AHA/ASA guidelines do not discuss preoperative DAPT for ICA occlusion (92–94). The evaluation of the efficacy of preoperative antithrombotic therapies in reducing early recurrent ischemic events and whether promote the clinical outcomes of patients under endovascular intervention or bypass surgery will be an another worthwhile topic in the further studies.

In this review, we searched the PubMed database for “chronic internal carotid artery occlusion, endovascular intervention, and external Carotid-internal Carotid Bypass”. Nineteen studies were included, including seven studies on EC-IC bypass surgeries and twelve on endovascular treatments (consisting of six studies on CAS and six on hybrid surgeries). Although two earlier studies were released in 1985 and 1995, these studies were released after 2006. A total of eight studies were controlled studies, nine were single-arm studies and two were retrospective analysis studies. Among the eight controlled studies, endovascular treatment was performed in three studies (82, 83, 88) and EC-IC bypass was performed in five studies (21, 57, 58, 62, 95). Among the nine single-arm studies, endovascular treatment was performed in seven studies (14, 83, 86, 95–97) and EC-IC bypass was performed in two studies (61, 100). Endovascular treatment was performed in two retrospective analysis studies (101, 102) (See Table 1).

**Table 1:** Basic characteristics of included studies.

Author	Year	Type of studies	Surgery vs. medicine	Interventions
Lin et al. (83)	2008	Single-arm	54	CAS
Xia et al. (83)	2012	Controlled	21 vs. 41	CAS
Fan et al. (82)	2014	Controlled	18 vs. 22	CAS
Chen et al. (101)	2016	Retrospective	138	CAS
Lee et al. (96)	2016	Single-arm	42	CAS
Kao et al. (102)	2018	Retrospective	118	CAS
Shih et al. (87)	2013	Single-arm	3	Hybrid
Liu et al. (97)	2018	Single-arm	21	Hybrid
Zhang et al. (88)	2019	Controlled	30 vs. 35	Hybrid
Jiang et al. (98)	2019	Single-arm	42	Hybrid
Yan et al. (99)	2020	Single-arm	37	Hybrid
Yang et al. (14)	2020	Single-arm	55	Hybrid
Unknown (21)	1985	Controlled	663 vs. 714	EC-IC
Ishikawa et al. (95)	1995	Controlled	27 vs. 36	EC-IC
Ogasawara et al. (57)	2006	Controlled	98 vs. 98	EC-IC
Powers et al. (58)	2011	Controlled	97 vs. 98	EC-IC
Fiedler et al. (61)	2011	Single-arm	20	EC-IC
Marshall et al. (62)	2014	Controlled	13 vs. 16	EC-IC
Komatani et al. (100)	2018	Single-arm	57	EC-IC

### Vascular recanalization rate

In two studies, EC-IC procedures reported a 96% recanalization rate. Possibly due to surgery to bypass diseased vessels and reconnect healthy ones without opening the occluded vessels (21, 62). Even if the vessel is completely occluded or if the calcification is very severe, postvascular flow is not affected. Five studies (82, 83, 96, 101, 102) on CAS reported the vascular recanalization rate. Compared with EC-IC, the recanalization rate of CAS is relatively low, possibly because the thrombus is hard and the micro guide wire cannot smoothly penetrate the thrombus. The recanalization rate of CAS may also be low because vascular calcification is severe, and the vascular stent cannot be successfully inserted. There was an increase in vascular recanalization rates with hybrid treatment. Six studies (14, 87, 88, 97–99) showed that vascular recanalization rates were increased when compared to that of CAS. Since carotid endarterectomy was performed prior to stent placement, most of the thrombus and atherosclerotic plaque were removed during carotid endarterectomy, thus allowing the guidewire to easily pass (See Table 2).

**Table 2:** Therapeutic effects and post-surgical complications of included studies.

Author	Interventions	Number	Vascular Recanalization Rate	Mortality Rate	Neurological function	Rate of Re-occlusion and Stroke
Lin et al. (83)	CAS	54	65%	4%	(−)	4%
Xia et al. (83)	CAS	21	(−)	4.8%	Improve	28.6%
Fan et al. (82)	CAS	18	100%	0%	(−)	0%
Chen et al. (101)	CAS	138	61.6%	(−)	(−)	(−)
Lee et al. (96)	CAS	42	69%	(−)	(−)	(−)
Kao et al. (102)	CAS	118	59.%	3.4%	(−)	16.1%
Shih et al. (87)	Hybrid	3	100%	0%	Improve	0%
Liu et al. (97)	Hybrid	21	71.4%	0%	(−)	0%
Zhang et al. (88)	Hybrid	30	100%	0%	Improve	0%
Jiang et al. (98)	Hybrid	42	83.3%	0%	(−)	6.7%
Yan et al. (99)	Hybrid	37	81.1%	0%	Improve	5.4%
Yang et al. (14)	Hybrid	55	78.2%	0%	Improve	1.8%
Unknown (21)	EC-IC	663	96%	0.6%	(−)	2.5%
Ishikawa et al. (95)	EC-IC	27	(−)	37%	No Improve	14.8%
Ogasawara et al. (57)	EC-IC	98	(−)	(−)	(−)	(−)
Powers et al. (58)	EC-IC	97	(−)	(−)	(−)	15%
Fiedler et al. (61)	EC-IC	20	(−)	(−)	Improve	(−)
Marshall et al. (62)	EC-IC	13	96%	30.8%	No Improve	15.4%
Komatani et al. (100)	EC-IC	57	(−)	(−)	(−)	28%

In addition, Chen et al. analyzed the independent factors associated with surgical recanalization rates by reviewing 138 CICAO patients who underwent the endovascular recanalization technique. It was concluded that the absence of previous neurological events, unpunctured stump, reconstruction of the distal ICA by contralateral injection, and distal ICA reconstruction with communication or ophthalmic segments were identified as independent negative predictors of the success of the CAO endovascular recanalization technique (101).

### Neurological function

Currently, the Modified Rankin Scale (mRS) and National Institute of Health Stroke Scale (NIHSS) are commonly used to assess neurological function. The mRS evaluates brain nerve function through the patient's clinical symptoms. It is divided into seven grades, ranging from asymptomatic to death (0–6 points) (103). The NIHSS evaluates neurological function in patients with stroke using fifteen items (104). Of the twelve included studies on endovascular treatment, a total of five studies mentioned neurological function after surgery. Xia et al. (83), Zhang et al. (88) and Yang et al. (14) evaluated the neurological function of patients by mRS, and the results showed improved function, and the effect was obvious when compared with that of the drug treatment group. ShiH et al. (87) and YAN et al. (99) reported that, according to NIHSS, endovascular therapy is helpful in the neurological recovery of CICAO patients. Only three studies on EC-IC bypass have reported neurological function. Ishikawa et al. designed a controlled trial comparing EC-IC bypass to medication (95). After two years of follow-up, there was no difference in neurological recovery between the two groups. Marshall et al. also shared the same point (62). Only the Fiedler et al. report showed a significant improvement in all areas of cognition twelve months after surgery (61) (See Table 2).

### Mortality rate

Only three studies have reported EC-IC bypass-related mortality rates of 0.6%, 37.0%, and 30.8%. Ten of the twelve studies on endovascular treatment reported mortality. The mortality rate of hybrid surgery is 0%, which is significantly better than that of other treatments. The reason may be the use of inconsistent methods to statistically measure mortality in the studies, some assessed perioperative mortality, or mortality at 2 or even 5 years after follow-up. However, overall, patients receiving endovascular treatment had significantly lower mortality (See Table 2).

### Reocclusion and stroke

EC-IC surgery is generally performed on the shallow temporal artery to supply cerebral blood flow, and its blood flow and perfusion are lower than those of the internal carotid artery, so the probability of stroke is relatively large. Five of these studies showed that the proportions of vascular reocclusion and stroke were 2.5%, 14.8%, 15%, 15.4%, and 28%, respectively (21, 58, 62, 95, 100). Although CAS can occlude open blood vessels, it does not remove endovascular plaque, and plaque inside or around the stent can easily cause thrombosis, so the proportion of vascular reocclusion and stroke will be relatively high. Four studies showed that the proportions of vascular reocclusion and stroke after CAS were 4%, 28.6%, and 0%, 16.1%, respectively (81–83, 100). Due to the removal of endovascular plaque in hybrid surgery, the proportions of vascular reocclusion and stroke are significantly reduced. Three (87, 88, 97) of six studies showed a proportion of 0%, while the proportions in the remaining three (14, 98, 99) were 6.7%, 5.4%, and 1.8% (See Table 2).

### Other complications

Other complications included cerebral hemorrhage, reperfusion injury, carotid artery cavernous sinus fistula, recurrent laryngeal nerve injury, pseudoaneurysm, etc. In a study on CAS, Lin et al. reported one carotid-cavernous sinus fistula (84), and Kao et al. reported four cases of cerebral hemorrhage (102). In a hybrid surgery study, Zhang et al. reported one cerebral hemorrhage and one recurrent laryngeal nerve injury (88); Yang et al. reported one reperfusion injury and one recurrent laryngeal nerve injury (14). In the study of EC-IC, Powers (58) reported 12 other complications: 4 TIAs, 2 intracranial hematomas, 2 seizures, 1 myocardial infarction, 1 case of respiratory disease, 1 case of hypotension and 1 wound infection. The occurrence of complications is closely related to the method of surgery. During endovascular treatment, the micro guidewire may cause vascular intimal injury, including a carotid cavernous sinus fistula, carotid-vascular perforation, and arterial dissection (6, 73). EC-IC bypass causes perfusion damage due to a mismatch in blood flow.

## Conclusion

Overall, EC-IC bypass is characterized by high recanalization and fewer complications. However, the mortality rate was relatively high, and the incidence of stroke was not reduced when compared with that in the medication group. In addition, the neurological function of the patients did not improve significantly after surgical treatment, and the clinical treatment effect was not obvious. Compared with EI-IC bypass, CAS has a good effect on nerve function, but the vascular recanalization rate is not high. The main reason is that long vascular lesions or the vascular intima have calcification and organization, and the guidewire has difficulty crossing the occluded segment. Furthermore, nonresidual obstruction prevents the guidewire from entering the true lumen and increases the risk of vascular damage. For hybrid surgery, the vascular recanalization rate and neurological improvement were satisfactory. The combination of carotid stenting and endarterectomy in hybrid surgery removes plaque and the thrombus well. Moreover, placing the stent under direct vision reduces vascular damage to a greater extent. Another advantage is that distal embolization is also prevented in hybrid surgery because retrograde flow can flush out the debris at the arteriotomy site. Therefore, hybrid surgery is a relatively satisfactory treatment option for CICAO.

At present, in the study of CICAO treatment, there are several studies on the comparison between surgical treatment and drug treatment, but studies on the comparison between different surgical methods are still relatively few. The number of studies selected in this paper was limited, the purpose and method of each study were different, and there was heterogeneity. In addition, the collection of study data, surgical technique proficiency, and operator experience also have an impact on the consistency of the study results. Currently, there are no clear criteria for the selection of CICAO surgical procedures. This paper provides ideas and reference for the choice of treatment through the statistical, comparative and analysis of the results of several studies. EC-IC bypass seems to be a better option if the occlusion range is large and the accumulated vessels are long. If the occlusion site is located in the skull and the occlusion range is small, CAS is a good choice. Hybrid surgery provides better treatment if the occlusion is located at the beginning of the internal carotid artery. However, the number of studies selected in this paper is limited and not representative, and more studies are still needed to determine the utility of this surgical method. Therefore, we hope that more studies can compare the outcomes of different surgical methods in patients with CICAO to provide a better treatment reference for future clinical work.
