# Supplementary material for: Spotlight on clinical strategies of Chronic Internal Carotid Artery Occlusion: Endovascular interventions and external-intracarotid bypasses compared to conservative treatment
Source: Front Surg. 2022 Nov 8;9:971066. doi: 10.3389/fsurg.2022.971066 (PMC9679017; doi:10.3389/fsurg.2022.971066)
Supplement: Supplementary file 1 [file Table1.docx]

**Supplementary Table 1. Abbreviation in this review.**

| **CICAO** | **Chronic internal carotid artery occlusion** |
| --- | --- |
| **EC-IC** | **External carotid-internal carotid** |
| **ICAO** | **Internal carotid artery occlusion** |
| **AICAO** | **Acute internal carotid artery occlusion** |
| **TIA** | **Transient ischemic attack** |
| **EEG** | **Electroencephalogram** |
| **TCD** | **Thermal conductivity detector** |
| **MCA** | **Middle cerebral artery** |
| **STA-MCA** | **Superficial temporal artery-middle cerebral artery** |
| **STA-SCA** | **Superficial temporal artery-superior cerebellar artery** |
| **OA-PICA** | **Ophthalmic artery-posterior inferior cerebellar artery** |
| **CAS** | **Carotid stenting** |
| **CEA** | **Carotid endarterectomy** |
| **TIA** | **Transient ischemic attacks** |
| **DAPT** | **Dual antiplatelet therapy** |
| **SAPT** | **Single antiplatelet therapy** |
| **AHA** | **American Heart Association** |
| **ASA** | **American Stroke Association** |
